# Avaliação da Função Renal em Pacientes com Insuficiência Cardíaca

**DOI:** 10.36660/abc.20210467

**Published:** 2021-08-09

**Affiliations:** 1 Universidade de São Paulo Faculdade de Medicina Instituto do Coração do Hospital das Clínicas São PauloSP Brasil Instituto do Coração do Hospital das Clínicas da Faculdade de Medicina da Universidade de São Paulo, São Paulo, SP - Brasil; 2 University Hospital Antwerp Antwerp Bélgica University Hospital Antwerp, Edegem, Antwerp - Bélgica

**Keywords:** Insuficiência Cardíaca/complicações, Doença Renal/complicações, Prognóstico, Prevenção e Controle, Fatores de Risco, Hipertensão, Diabetes Mellitus, Hipovolemia

Insuficiência cardíaca é uma doença sistêmica que pode levar à disfunção de outros órgãos e sistemas fisiológicos, que por sua vez podem trazer sintomas e influir negativamente no prognóstico dos pacientes.^1^^,^^2^ O comprometimento da função renal é uma das disfunções que podem ocorrer.^2^^,^^3^

A prevenção, o diagnóstico e o tratamento dessa complicação são fundamentais para o tratamento de pacientes; estratégias de categorização desse comprometimento foram criados e são úteis para a prática clínica.^1^

Um primeiro passo seria estudar a experiência local para estimar a frequência, características clínicas e a gravidade do comprometimento renal. Admite-se que a lesão renal aguda possa variar de acordo com a geografia e características socioeconômicas regionais.^4^ O estudo retrospectivo baseado em registros hospitalares de um hospital de referência no nordeste brasileiro comparou dois métodos de avaliação do comprometimento renal.^5^

Foi estudada^5^ uma amostra de 81 pacientes, sendo 53% de homens e 47% de mulheres, internados no Hospital com diagnóstico de insuficiência cardíaca, com média de idade de 67 anos; 16 pacientes haviam sofrido infarto do miocárdio recente. Diagnosticou-se lesão renal aguda em 50/81 pacientes; houve 16/50 (32%) de óbitos entre pacientes com lesão renal aguda e 3/31 (9,68%) de óbitos entre pacientes sem lesão renal aguda. Os autores verificaram que o critério KDIGO (*Kidney Disease: Improving Global Outcomes*) orientou o diagnóstico em 61,7% dos casos; o critério AKIN (*Acute Kidney Injury Network*) não foi sugestivo de lesão renal em 14% dos casos. Os autores concluíram que nessa amostra não se evidenciou uma relação clara entre a condição cardíaca e a lesão renal; os critérios empregados não demonstraram, nessa amostra, diferença na orientação diagnóstica.

A prevenção é fundamental no tratamento de pacientes. Prevenção de lesão renal aguda faz parte do tratamento de pacientes com insuficiência cardíaca e inclui: controle de fatores que contribuem para a ocorrência de disfunção renal e cardíaca (como, por exemplo, diabetes melito e hipertensão arterial); prevenção e controle de fatores agravantes da função renal, como hipovolemia, hipotensão e uso de medicamentos potencialmente nefrotóxicos; prevenção e tratamento de fatores agravantes da insuficiência cardíaca, como hipovolemia, variação abrupta de pressão arterial, isquemia e uso de medicamentos que prejudicam a função cardíaca, entre outros. O tratamento apropriado da insuficiência cardíaca com medicamentos eficazes influem favoravelmente na função renal.

No caso de lesão renal aguda estabelecida, o diagnóstico na sua fase inicial pode ser crucial e os critérios diagnósticos podem auxiliar neste sentido. Entre eles, o mais usado na prática clínica é a elevação de creatinina sérica igual ou superior a 0,3 mg/dL, que se reconhece como associada ao prolongamento de internações e elevação da mortalidade. Entretanto, não há uma relação linear entre a creatinina sérica e a taxa de filtração glomerular; influem estado metabólico, idade, sexo, raça, estado nutricional.^6^

Uma forma de compensar essas limitações foi o desenvolvimento de equações para o cálculo da taxa de filtração glomerular usando variáveis como sexo, idade, raça e área de superfície corporal. As equações mais comumente usadas são Cockcroft-Gault, MDRD (Modificação da dieta na doença renal) e CKD-EPI (Chronic Kidney Disease Epidemiology Collaboration). Apesar de mais precisos, eles também enfrentam limitações em populações especiais, como pacientes idosos, com baixo peso, obesos e diabéticos.

Os critérios usados na prática clínica usam a creatinina (AKIN), estimativas da taxa de filtração glomerular (KDIGO) ou a combinação de ambos (RIFLE) em associação com outras variáveis como a albuminúria. Outros marcadores de função renal incluindo a isoforma da molécula-1 de lesão renal (KIM1), N-acetil-β-D-glicosaminidase (NAG), interleucina 18, cistatina C, lipocalina associada a gelatinase neutrofílica (NGAL) e exosomas urinários são no momento tema de investigação.^7^

## References

[1] 1. Thomas ME, Blaine C, Dawnay A, Devonald MA, Ftouh S, Laing C, et al. The definition of acute kidney injury and its use in practice. Kidney Int. 2015 Jan;87(1):62-73. doi: 10.1038/ki.2014.328. 10.1038/ki.2014.328 25317932

[2] 2. Villacorta H, Villacorta AS, Villacorta LSC, Xavier AR, Kanaan S, Rohen FM,et al. Worsening Renal Function and Congestion in Patients with Acute Heart Failure: A Study with Bioelectrical Impedance Vector Analysis (BIVA) and Neutrophil Gelatinase-Associated Lipocalin (NGAL). Arq Bras Cardiol. 2021 Apr;116(4):715-24. 10.36660/abc.20190465 PMC8121398 33886716

[3] 3. Leite AM, Gomes BFO, Marques AC, Petriz JLF, Albuquerque DC, Spineti PPM, et al. Acute Cardiorenal Syndrome: Which Diagnostic Criterion to Use And What is its Importance for Prognosis? Arq Bras Cardiol. 2020 Jul;115(1):127-33. 10.36660/abc.20190207 PMC8384316 32813824

[4] 4. Mehta RL, Burdmann EA, Cerdá J, Feehally J, Finkelstein F, García-García G, et al. Recognition and management of acute kidney injury in the International Society of Nephrology 0by25 Global Snapshot: a multinational cross-sectional study. Lancet. 2016 May 14;387(10032):2017-25. doi: 10.1016/S0140-6736(16)30240-9. Erratum in: Lancet. 2016 May 14;387(10032):1998. 10.1016/S0140-6736(16)30240-9 27086173

[5] 5. Nascimento GVR, Brito HCD, Lima CEB. Type 1 Cardiorenal Syndrome in Decompensated Heart Failure Patients in a Low-Income Region in Brazil: Incidence of Acute Kidney Injury (AKIN and KDIGO Criteria), Need for Dialysis and Mortality. Arq Bras Cardiol. 2021; 117(2):385-391. doi: https://doi.org/10.36660/abc.20200097. 10.36660/abc.20200097 PMC8395783 34495237

[6] 6. Blair JE, Pang PS, Schrier RW, Metra M, Traver B, Cook T, et al. EVEREST Investigators. Changes in renal function during hospitalization and soon after discharge in patients admitted for worsening heart failure in the placebo group of the EVEREST trial. Eur Heart J. 2011 Oct;32(20):2563-72. doi: 10.1093/eurheartj/ehr238. 10.1093/eurheartj/ehr238 21785107

[7] 7. Lassus JP, Nieminen MS, Peuhkurinen K, Pulkki K, Siirilä-Waris K, Sund R,et al. FINN-AKVA study group. Markers of renal function and acute kidney injury in acute heart failure: definitions and impact on outcomes of the cardiorenal syndrome. Eur Heart J. 2010 Nov;31(22):2791-8. doi: 10.1093/eurheartj/ehq293. 10.1093/eurheartj/ehq293 20801926

