# Supplementary material for: Avaliação da Função Renal em Pacientes com Insuficiência Cardíaca
Source: Arq Bras Cardiol. 2021 Aug 9;117(2):392–3. [Article in Portuguese] doi: 10.36660/abc.20210467 (PMC8395803; doi:10.36660/abc.20210467)
Supplement: Translation

# Translation: Assessment of Renal Function in Patients with Heart Failure

**Affiliations:** 1 São PauloSP Brazil Instituto do Coração do Hospital das Clínicas da Faculdade de Medicina da Universidade de São Paulo, São Paulo, SP - Brazil; 2 Antwerp Belgium University Hospital Antwerp, Edegem, Antwerp - Belgium

**Keywords:** Heart Failure/complications, Kidney Diseases/complications, Prognosis, Prevention and Control, Risk Factors, Hypertension, Diabetes Mellitus, Hypovolemia

Heart failure is a systemic condition that may lead to impairment of many organ systems and physiological functions that may cause symptoms and may have a negative influence in the prognosis of the patients.^1^^,^^2^ Compromising renal function may be one of the ailments of patients with heart failure.^2^^,^^3^

Prevention, diagnosis and treatment of this complication are key issues in patient care; strategies of categorization of acute renal lesions have been developed and are helpful in clinical practice.^1^

A first step would be to study local experience to estimate its frequency, clinical characteristics and evaluation of severity. Causes of acute kidney injury were recognized to vary by country and economic status.^4^ This important step was provided in a retrospective study based on hospital charts of a referral hospital in a Brazilian northeast state capital to address the comparison of two methods of evaluating renal dysfunction.^5^

They studied^5^ a sample of 81 patients admitted to the Hospital, diagnosed with heart failure (16 of them with recent myocardial infarction), mean age 67 years, men (53%) and women (47%). Acute kidney injury was diagnosed in 50/81 patients; mortality was 16/50 (32%) in patients with acute kidney injury and 3/31 (9,68%) in patients without acute kidney injury. Dialysis was performed in three patients with acute kidney injury. The authors found that the KDIGO score (Kidney Disease: Improving Global Outcomes) indicated renal injury in 61,7% of the cases; the AKIN (Acute Kidney Injury Network) criteria did not indicate acute renal lesion in 14% of the patients. The authors concluded that a relationship between the cardiac conditions in this study sample did not demonstrate a clear relationship with acute kidney lesions; the scores did not demonstrate a significant difference in performance for guidance in the diagnosis of acute kidney lesions.

Prevention is a key step in patient care. Prevention of acute kidney injury may be part of the care of patients with heart failure and include: control of contributing factors to the occurrence and development of both renal and cardiac dysfunctions (such as diabetes mellitus and arterial hypertension); prevention and control of factors aggravating kidney function such as hypovolemia, hypotension and use of nephrotoxic agents; prevention and control of factors aggravating heart function such as hypo- and hypervolemia, acute blood pressure abnormalities, ischemia and use of drugs that could impair the heart function, and others; appropriate treatment of heart failure with disease-modifying therapies and, ideally, use of therapies that could positively impact the renal function.

In the event of established acute kidney injury, early diagnosis or prompt diagnosis may be crucial and the use of appropriate diagnostic criteria play a central role. One of the most used markers in medical practice is serum creatinine and elevation equal to or greater than 0.3 mg/dl has been associated with increased risk of hospital death and prolonged hospitalization. However, the non-linear relationship between creatinine and glomerular filtration rate and the influence of factors such as metabolic status, age, gender, race, and nutritional status limit an accurate application in clinical practice.^6^

A way to compensate for these limitations was the development of equations for calculating glomerular filtrating rate using variables such as sex, age, race, and body surface area. The most commonly used equations are Cockcroft-Gault, MDRD (Modification of Diet in Renal Disease) and CKD-EPI (Chronic Kidney Disease Epidemiology Collaboration). Despite being more accurate, they also face limitations in special populations such as elderly, underweight, obese and diabetic patients.

Scores frequently used in clinical practice use either creatinine (AKIN), glomerular function estimates (KDIGO) or a combination of both (RIFLE), in association with other data, such as albuminuria. Other markers of renal function including kidney injury molecule 1 (KIM1), isoform 1, N-acetyl-β-D-glycosaminidase (NAG), interleukin 18, cystatin C, neutrophil gelatinase-associated lipocalin (NGAL), and urinary exosomes are currently under investigation.^7^
